# New products

**DOI:** 10.1155/S1463924693000203

**Published:** 1993


Journal of Automatic Chemistry, Vol. 15, No. 4 (July–August 1993), pp. 151–157

### New products

### Tray dryer

The Dura-Stop Tray Dryer offers automated operation of the entire freeze drying process, as well as protection for the system and product in the event of a power failure. Options include −70°C shelf temperature, an isolation valve, a Control Workstation with Lyphoware software, and hydrogen peroxide vapor sterilization.

The designed Dura-Dry condenser module of the Dura-Stop Stoppering Tray Dryer includes stainless-steel vacuum tubing assemblies for improved vacuum pulldown and leak rate. The new condenser door design allows for easy access to the titanium condenser and eliminates the need for vacuum grease. The access door allows for easy checking of vacuum pump oil level and quality. A convenient drain makes vacuum pump oil changes easy.

*Details from Joe Brendle, Division Manager, Life Science Division, FTS Systems, Inc., PO Box 158, Route 209. Stone Ridge, New York 12484, USA. Tel.: 800 251 1531 (US only) or fax: 914 687 7481.*

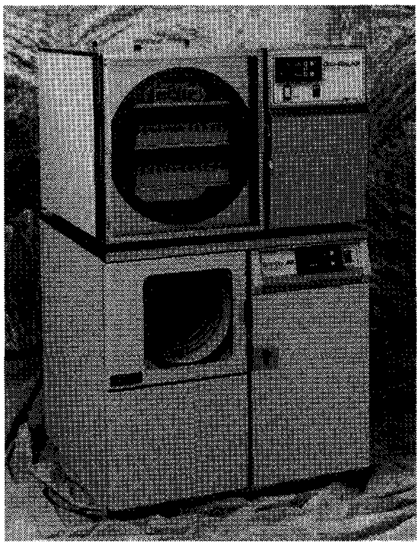

*Figure 1. Dura-Stop Tray Dryer (FTS Systems).*

### Mercury and hydride detection

Perkin-Elmer's FIAS-THGA Coupling Kit will link the FIAS 400 or FIAS 200 Flow Injection System to the transversely heated graphite furnace (THGA) in the 4100ZL AAS. With this instrument configuration the automated FI-MHS system acts as the sample preparation and delivery station for graphite furnace AAS, automating on-line element preconcentration in the graphite tube. Sample volumes of several millilitres can then be applied to graphite furnace AAS, improving detection limits for the hydride-forming elements and mercury by a factor of between 50 and 500.

As a result, element concentrations of a few ng/l can routinely be determined in non-polluted water or medical samples, without time-consuming sample preconcentration. Matrix interference and nonspecific absorption are reduced, since matrix components are removed in the flow injection system.

*Further information from Perkin-Elmer Ltd, Post Office Lane, Beaconsfield, Buckinghamshire HP9 1QA, UK. Tel.: 0494 679026; fax: 0494 679064.*

### Autosampler control software

ATI Unicam's 240 diode array detector, 200 quaternary pump and their 230 autosampler can be controlled using Windows 3.1 based control software. Any single or dual combination of these three instruments can be controlled with a clear and simple upgrade to a full system. Once computer control of all three instruments is achieved, the system can be further upgraded to a method development chromatograph. The control software forms the core of this system by trading instrumental methods and data with two expert systems. The Diamond system optimizes solvent selectivity in reversed phase LC using only 10 experiments by tracking peaks across an isoeluotropic plane. No standards are necessary as interpretation is undertaken entirely by the software which develops chromatographic methods by using Iterative Target Transfer Factor Analysis (ITTFA) in conjunction with the control software.

*Further information from Paul Carter, ATI Unicam, York Street, Cambridge CB1 2PX, UK. Tel.: 0223 358866; fax: 0223 312764.*

### Robotic system

The Perkin-Elmer Thermal Analysis 7 Series/UNIX DSC 7 RS Robotic System operates under the 7 Series/UNIX software platform with industry-standard DECpc 433dx LP personal computer systems for automated Differential Scanning Calorimetry (DSC) testing. The system permits analysis of up to 48 samples of the same or different type, for increased efficiency and lower cost per analysis.

An add-on autosampler designed to be used with the DSC 7 Differential Scanning Calorimeter, the Robotic System can use multiple experimental methods and run multiple methods on a single sample. Samples may be analysed using any previously stored method in user-defined sequence. The system also has multiple ramping capability in a single method, as well as automatic peak, glass transition and onset analysis.

*Further information from Perkin-Elmer (above).*

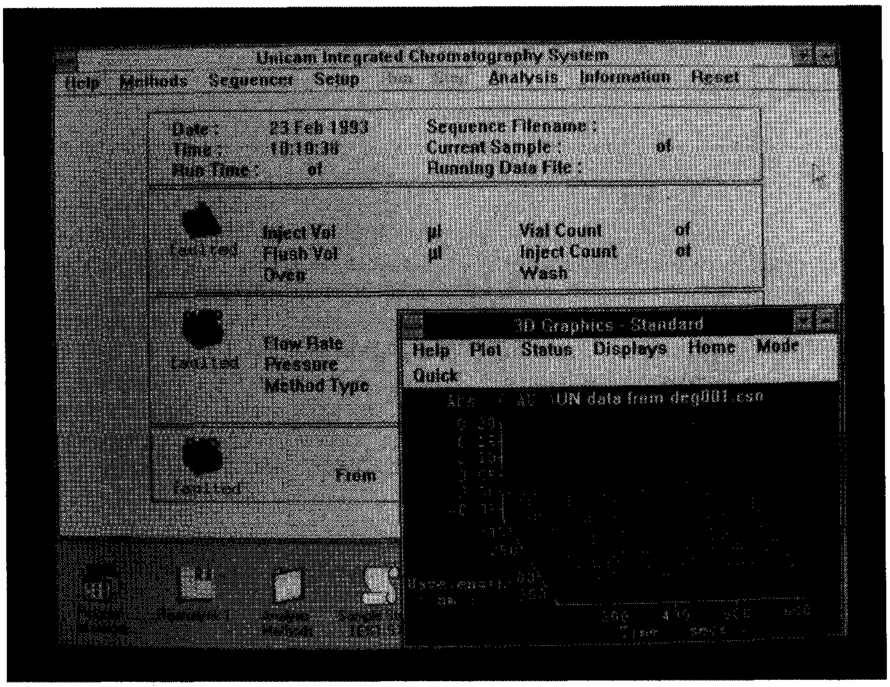

*Figure 2. Autosampler control software (ATI Unicam); see previous page.*

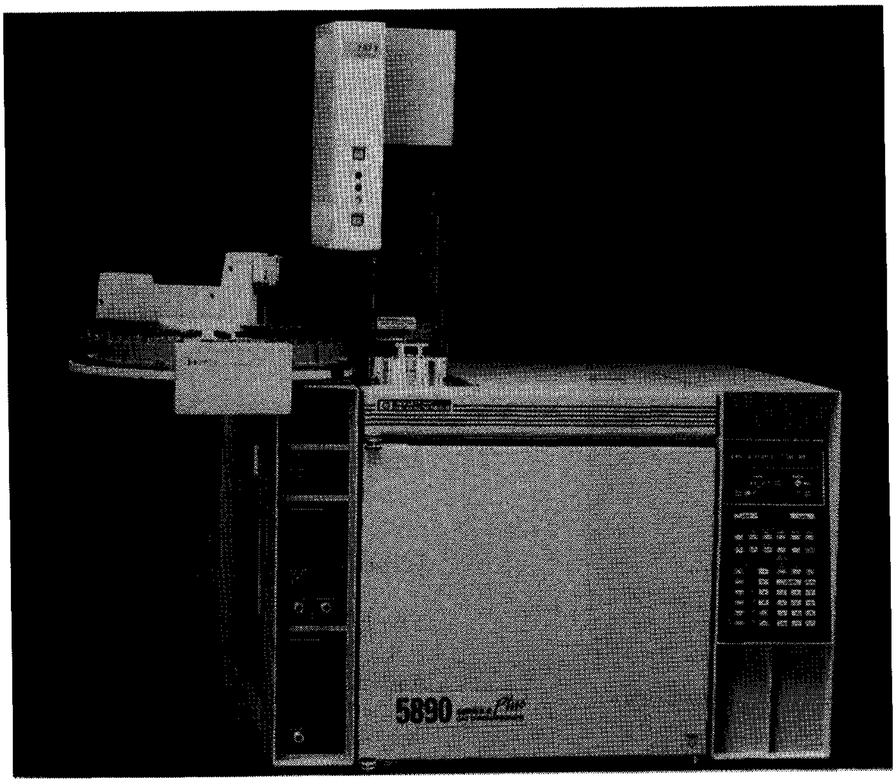

*Figure 3. The HP 5890 Series II Plus, with up to six channels of electronic pressure control, provides precise digital control of all GC gases, both inlet and detector. When combined with an HP autosampler and ChemStation, totally automated gas chromatography is possible.*

### GC with electronic pressure control

Hewlett-Packard's 5890 Series II Plus gas chromatograph (GC) offers expanded electronic pressure control (EPC) capability. The addition of four auxiliary EPC channels provides precise digital control of all GC gases for both inlets and detectors.

Users of the HP 5890 Series II Plus will have the same level of digital programming capability and ease of controlling gas flows as they are accustomed to in column oven control. This results in enhanced GC performance and precision, increased productivity and reduced operating costs.

Two EPC channels are dedicated to inlets and operate in several modes, including constant pressure, constant flow in capillary columns, pressure programming and vacuum compensation for use with mass spectrometers. The four auxiliary EPC channels can be used to optimize performance for a variety of applications. They provide control for carrier gases, reagent gases and detector make-up and fuel gases. They also control flows from sample introduction devices such as the HP 7694 headspace sampler and purge-and-trap units.

Pressure programming can reduce sample discrimination and decomposition by controlling sample expansion volume; and it can facilitate efficient transfer of the sample on to the column. This can also result in higher sensitivity by allowing larger injection volumes to be used in splitless injection. Precise flow control of detector gases improves the performance and stability of the detector.

EPC reduces retention times, lowers elution temperatures and reduces thermal stress on columns. It also increases system throughput by programming the pressure at the column head to compensate for increasing gas viscosity during an oven temperature program.

EPC also permits faster, more efficient transfer of the sample from external sampling devices (the HP 7694 headspace sampler for example). With EPC, the operator spends less time on method set-up, so the system as a whole is easier to use. The system can be reset to previously-stored parameters quickly and easily.

EPC reduces wastage by automatically slowing the flow of expensive reagent, carrier and fuel gases during stand-by periods. The EPC Gas Saver mode reduces carrier gas consumption during off hours and controls split vent flow.

When used with the HP 3365 Series II ChemStation (Revision 3.33), single-point control is available over all GC parameters including split ratio and split vent flow.

The digital control provided by EPC means that each chromatographic run is performed under the same set of conditions. Reproducibility is therefore improved from run to run, from instrument to instrument and from operator to operator.

All GC parameters are logged automatically, and an audit trail of data and test conditions help the laboratory comply with good laboratory practice (GLP) requirements.

The HP 5890 Series II Plus with six-channel EPC, combined with the HP 7673 automatic injection/sampler system and the HP 3365 Series II ChemStation, provides a fully automated GC system for research and quality control. As such, it will have applications in petrochemical and chemical manufacturing plants, in food and pharmaceutical production, in environmental laboratories and other areas where fully automated gas chromatography is required.

*Enquiries to Verena Haller, Hewlett-Packard SA, 150 Route du Nant-d'Avril, CH 1217 Meyrin (GE) 2, Switzerland.*

### Professional chemists live longer

Professional chemists apparently live longer than average and, in general, die less often from cancer. Research into the causes of death of professional chemists, published in the May 1993 issue of the *American Journal of Industrial Medicine*, shows, however, that chemists contract certain cancers more frequently than expected.

In the 25-year study, conducted by the Royal Society of Chemistry, the causes of the deaths of 4012 chemists (out of a membership of 14 884) were analysed.

The results show that while chemists have a higher-than-expected rate of death from: lymphatic cancers; leukaemias; gastrointestinal cancers; duodenal, kidney, and skin cancers; their overall mortality from cancer is less than in similar professional classes.

The results also show a larger than expected incidence of mental disorders and diseases of the nervous system.

### Micro-volume flame analysis

Multi-elemental analysis on a limited volume sample by flame atomic absorption has always caused problems. Shimadzu has introduced a micro-volume flame analysis system with their new AA-6501 Series Atomic Absorption Spectrophotometers, which uses a typical 60 μl sample volume. The technique can be fully automated with the use of the ASC6000 semi-intelligent preparation station. Standard curves can be produced from a single stock standard, essential matrix modifiers added automatically and analysis performed on up to 60 samples. This micro-volume technique is also useful when sample concentration is necessary, it is easier to concentrate 10 ml to 1 ml than 100 ml to 10 ml.

*Further information from V. A. Howe & Co. Ltd, Beaumont Close, Banbury, Oxfordshire OX16 7RG, UK. Tel. 0295 252600; fax: 0295 26896.*

### Temperature checks

The Thermapen pocket thermometer gives a clear digital reading of temperatures from −50°C to +300°C, with an accuracy of better than 0.3°C. When not in use, the hinged probe simply folds away so the Thermapen can be slipped into a pocket until next required. Even corrosive substances, including acids, can be tested using the PTFE coated stainless-steel probe (an option, in addition to the standard stainless-steel probe).

Two different Thermapen pocket thermometers are available with either 0.1 or 1°C resolution. The Thermapen 1 accurately registers temperatures in the range −49.9 to +199.9°C and the Thermapen 5 between −50 and +300°C. All thermometers manufactured by Electronic Temperature Instruments are calibrated against laboratory standards, traceable via NAMAS Certification to British National Standards. Each instrument is supplied with a works Calibration Card and full, original NAMAS Calibration Certificates are available if required.

Each Thermapen is supplied with a detachable wrist-strap and 12V battery, which should give approximately 100 or 200 hours of use with the Thermapen 1 or Thermapen 5 respectively.

Electronic Temperature Instruments Ltd, which celebrates its tenth anniversary this year, has established an excellent reputation for the manufacture of high quality temperature instrumentation. ETI can supply accurate thermometers and probes to meet an enormous range of industrial and laboratory requirements.

*Details from Peter Webb, ETI Ltd, Southdownview Road, Broadwater Trading Estate, Worthing, West Sussex BN14 8NL, UK. Tel: 0903 202151; fax: 0903 202445.*

### Pipette tips

BIO 101 Inc.'s Triple Check tip allows visual confirmation of exact low volume measurements of enzymes and other expensive or critical liquids. The pipette tip fits most pipettors and is marked with graduations at precise volumes of 0.5, 1, 2, 3, 4, 5, 10, 50, 100, and 150 μl. The pipette tip will eliminate errors associated with viscosity differences among various reagents. The Triple Check tips

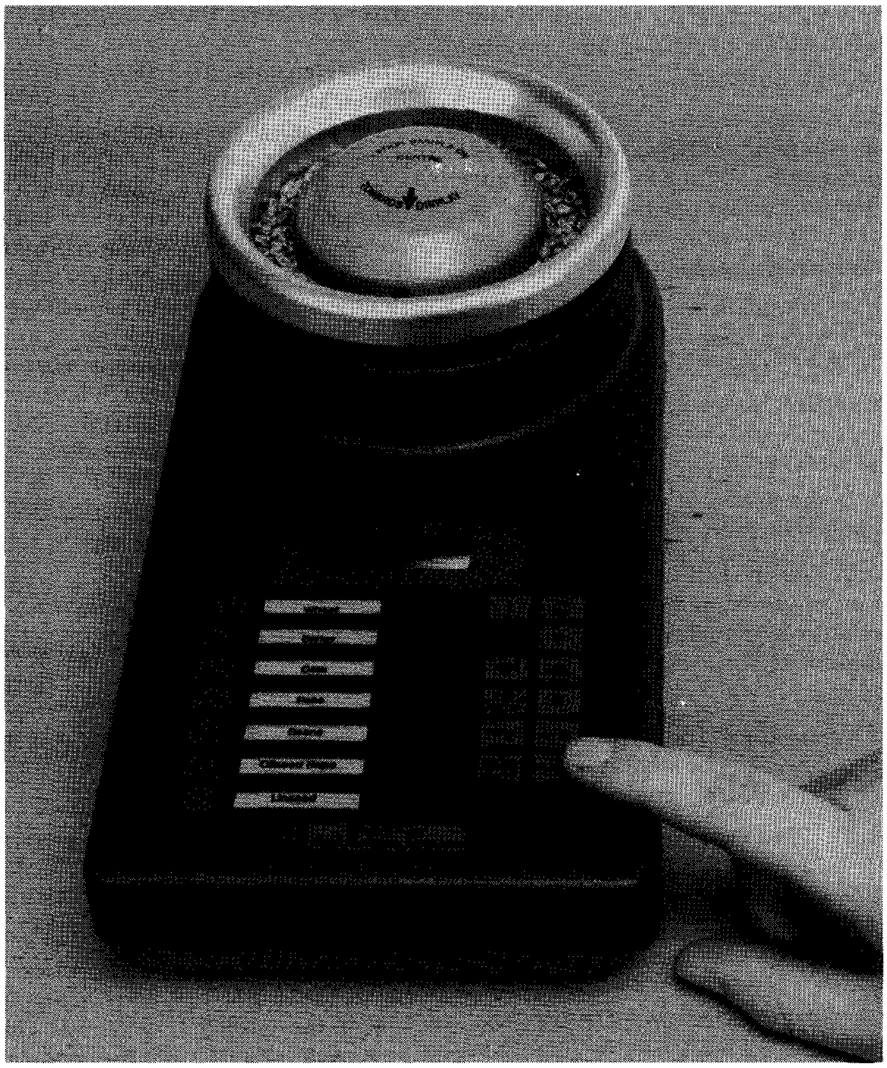

*Figure 4. Pre-calibrated for seven commodities the Sinar AP Moisture Analyser produces moisture results in 6 s using whole grain samples. Standard packages are available for both temperature and tropical agricultural commodities, including all types of cereal grains, oilseeds, pulses, nuts, tea, coffee, cocoa and spices. Calibrations can be individually adjusted when required to optimize the accuracy and a security password ensures that only authorized users are able to make calibration adjustments. The average moisture content of up to 254 samples can be measured in one second. Temperature (°C or °F) and bulk density (kg/hl or lb/bu) readings are also provided and results can be fed to a printer for a hard copy record. Details from Tecator AB, Box 70, S-263 21 Höganäs, Sweden. Tel.: 46 42 36 15 00; fax: 46 42 34 03 49.*

are available in racks of 96 tips, with or without aerosol barriers, sterile and non sterile.

*Details from BIO 101, Inc. 1060 Joshua Way, Vista, California 92083, USA. Tel.: 619 598 7299; fax: 619 598 0116.*

### AA applications

GBC Scientific Equipment offer an Atomic Absorption Applications Report which describes the performance of their GBC Atom Trap. This device consists of a slotted quartz tube which is positioned in the light path above an air-acetylene burner. The atoms are trapped in the tube and held in the light path for a longer period of time than normal.

*Copies from Orry Dugdale, GBC Scientific Equipment UK Ltd, 13 Frederick Sanger Road, The Surrey Research Park, Guildford Surrey GU2 5YD, UK. Tel.: 0483 304988; fax: 0483 303071.*

### Optical emission spectrometer

Perkin-Elmer's Optima 3000 is a computer-controlled, Inductively Coupled Plasma Optical Emission Spectrometer (ICP-OES). The 3000's detector, designed specifically for plasma emission spectroscopy, and patented optical system permit simultaneous measurement of spectral background and each analyte line. Sixty elements can be measured in less than 1 min at multiple wavelengths, with no loss of precision or sensitivity. The Optima 3000 includes over 5000 emission lines so that alternate interference-free lines can be selected for superior results.

The spectrometer combines detector, 40-MHz free-running RF generator with new power control system, and temperature-controlled plasma pneumatics in a free-standing, easily-serviced, one metre square package.

All instrument functions are computer-controlled, and specialized software stores all relevant operating

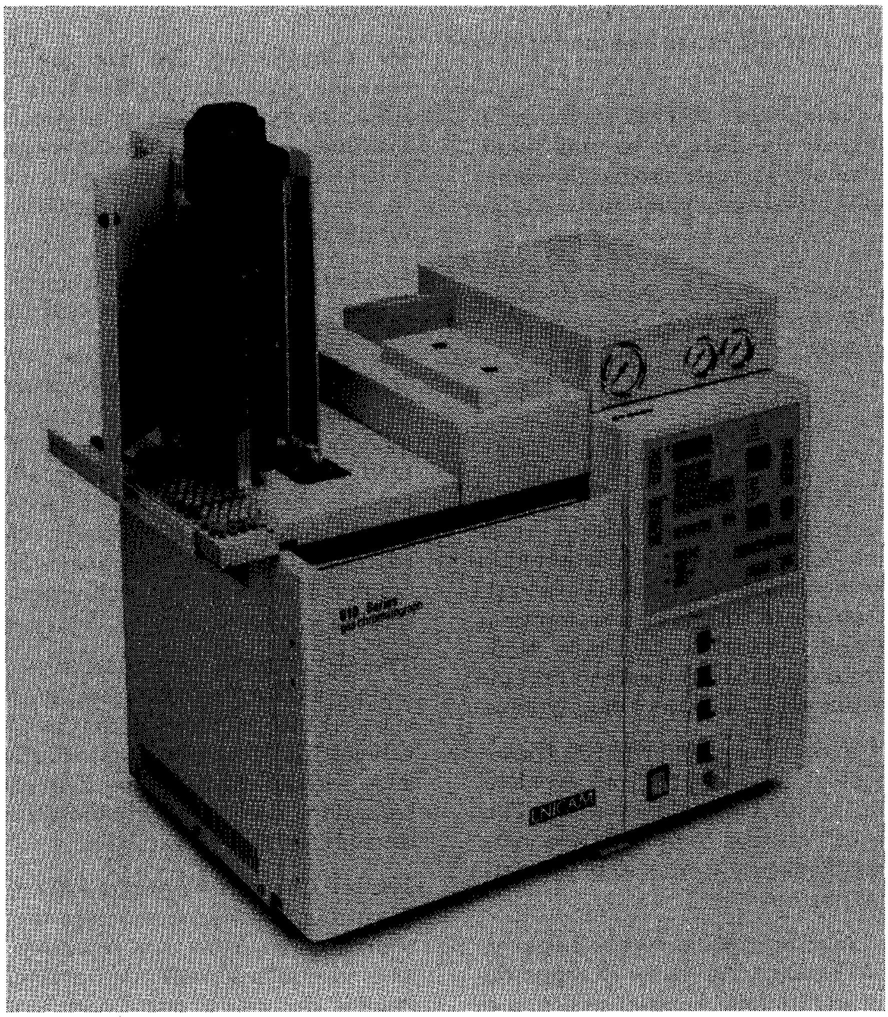

*Figure 5. ATI Unicam's nitrogen detector for the 610 Series gas chromatograph. The detector could find applications in environmental and drug-related analyses; it has a long life, high stability source which gives stable results. In addition, it has a source protection function, to reduce the possibility of poisoning from high levels of solvents. Details from ATI Unicam Ltd, York Street, Cambridge CB1 2PX, UK. Tel.: 0223 358866; fax: 0223 312764.*

parameters with an automated optimize function to simplify method development. The software stores the analytical results and provides post-analysis data processing of stored spectra.

*Details from Perkin-Elmer Ltd (above).*

### Sample changer

Radiometer Analytical's SAC90 provides automated analyses of a large number of samples. Depending on the analyses to be performed, the user can handle from six to 99 samples with the basic SAC90 version. The smaller the sample size, the more samples that can be analysed. It is possible to double or triple the sample capacity by adding up to two side tables.

If daily analyses are based on titrations, the user can choose between two control units in the TitraLab titration product range—the VIT90 Video Titrator or the TIM90 Titration Manager. If the user primarily performs pH or ion-selective measurements, the SAC90 can be controlled by two of the pH meters in the MeterLab product range—the PHM93 Refernce pH meter and the PHM95 pH/ION Meter.

The VIT90 Video Titrator and SAC90 combination allows up to six different methods to be run simultaneously in the same sample. The user also has the choice of separating the samples into six groups for analysis using different methods.

*More information from Lene Henriksen, Radiometer Analytical A/S, Krogshojvej 49, DK 2880 Bagsvaerd, Denmark. Tel.: 45 31 69 6311; fax: 45 4449 0011.*

### Network servers

Hewlett-Packard's ChemServer 4900 series family of network servers, are the first open-systems based server systems designed specifically for the analytical laboratory. HP ChemServers allow laboratory managers and chemists to perform a wide range of analytical networking, processing, reporting and communications tasks while improving adherence to regulatory requirements and the overall productivity of analytical laboratory data systems.

Using HP ChemLAN networking products, HP ChemServers can bring together information from Hewlett-Packard and other vendors' analytical data systems. This allows laboratories to use existing computers and data handling devices, enabling them to share and use data from various analytical instruments.

HP ChemServers are based on client/server technology. Rather than one system handling the range of analytical tasks associated with an application, client/server computing allows multiple computers on a network to perform tasks in parallel. As a result, computers on the network can perform the task for which they are best suited.

HP ChemServers are designed to work with existing laboratory instrument controllers and data systems. Specifically, they can enhance the productivity of HP ChemStations, DOS-based data systems from other vendors, HP's 3350A Laboratory Automation Systems and laboratory information management systems (LIMS). With a network architecture based on industry and *de facto* standards such as IEEE 802.3 and Transport Control Protocol/Internet Protocol (TCP/IP), HP ChemServers can also be connected to mainframe and business systems to provide complete system integration within a company-wide networking strategy. HP ChemServers use HP ChemLAN networking products, which makes it quick and easy to install and customize a network to accommodate the various instrument controllers within the laboratory.

HP ChemServers are designed to enhance a laboratory's ability to comply with Good Laboratory Practice (GLP), ISO 9000 and other regulatory guidelines. Since the HP ChemServer systems are password-protected, they provide secure access to data and resources on the laboratory network. The system administrator can restrict personnel from accessing sensitive data or working on unauthorized tasks. Additionally, the HP ChemServers' automated backup capability ensures accurate and consistent archiving.

HP ChemServers are true multitasking systems. For example, users can archive data from one instrument while they view incoming in-process data from another instrument. The HP ChemServer graphical user interface (GUI) makes the system easy to use, with common tasks such as processing or reviewing data performed by clicking on the relevant icon.

Hewlett-Packard offers a choice of HP ChemServer models, with two different systems currently available.

The HP ChemServer Model 4910, the Laboratory Network ChemServer, allows users to share information by functioning as a central archive for important laboratory information. It suggests Pascal, MS-DOS® and UNIX® based client computers, allowing laboratories to enhance the usefulness of their current data systems. This HP ChemServer is particularly suited for chemists and technicians who work with single-user systems and require additional resources for archiving data and the ability to connect to systems outside the laboratory.

The HP ChemServer Model 4910 allows users to share peripherals such as colour and black-and-white printers, allowing higher quality output at a lower cost and saving valuable bench space; automatically back up laboratory data, without needing to back up each workstation in the laboratory daily; and administrate the network easily through the HP VUE GUI.

The HP ChemServer Model 4920, the Data Analysis and Reporting ChemServer, brings together the processing, review, maintenance and reporting of chromatographic, mass spectral and multiple wavelength liquid chromatographic data on a single high performance laboratory workstation. This HP ChemServer is for laboratories that require higher productivity and need to meet stringent quality assurance (QA) and regulatory standards.

The HP ChemServer Model 4920 includes all the capabilities of the Model 4910 and also allows users to review data in a consistent fashion from different instruments and analytical techniques throughout a laboratory workgroup; improve QA by making it possible to work with up to 30 different quality control sample types, to specify detection and other limits and to generate a range of reports that help pinpoint quality problems within the analytical process; and generate an audit trail of all changes to methods and data.

*Details from Hewlett-Packard (above).*

### Royal Society Medals

The Council for the Royal Society has awarded the following medals for 1993:

**The Copley Medal** to Dr J. D. Watson, For.Mem.R.S., Director of the Cold Spring Harbor Laboratory, New York, USA, in recognition of his tireless pursuit of DNA, from the elucidation of its structure to the social and medical implications of the sequencing of the human genome.

**The Davy Medal** to Professor Jack E. Baldwin, F.R.S., Waynflete Professor of Chemistry in the University of Oxford, distinguished for his contributions to bio-organic chemistry, in particular to an understanding of the biosynthesis of beta-lactam antibodies.

**The Hughes Medal** to Professor G. R. Isaak, Professor of Physics in the University of Birmingham, in recognition of his pioneering use of resonant scattering techniques to make extremely precise measures of Doppler velocity shifts in the solar photosphere.

**The Leverhulme Medal** to Professor J. S. Rowlinson, F.R.S., Dr Lee's Professor of Chemistry in the University of Oxford, distinguished for this contributions to thermodynamics, in particular to an understanding of the physical chemistry of gas-liquid interfaces and surfaces.

